# Supplementary material for: A Comprehensive Assessment of Ultraviolet-Radiation-Induced Mutations in Flammulina filiformis Using Whole-Genome Resequencing
Source: J Fungi (Basel). 2024 Mar 20;10(3):228. doi: 10.3390/jof10030228 (PMC10971301; doi:10.3390/jof10030228)
Supplement: Supplementary file 1 [file jof-10-00228-s001.zip › Supplementary Material S8/KEGG annotation/out/64381550635650.os/KO/out_map/map05146.html]

KEGG PATHWAY: Amoebiasis - Reference pathway


|  |  |
| --- | --- |
| **Amoebiasis - Reference pathway** |  |

[
Pathway menu
| Organism menu
| Pathway entry
| Show description
| User data mapping
]

Entamoeba histolytica, an extracellular protozoan parasite is a human pathogen that invades the intestinal epithelium. Infection occurs on ingestion of contaminated water and food. The pathogenesis of amoebiasis begins with parasite attachment and disruption of the intestinal mucus layer, followed by apoptosis of host epithelial cells. Intestinal tissue destruction causes severe dysentery and ulcerations in amoebic colitis. Several amoebic proteins such as lectins, cysteine proteineases, and amoebapores are associated with the invasion process. The parasite can cause extraintestinal infection like amoebic liver abscess by evading immune response.

|  |  |
| --- | --- |
| Reference pathway | 100% |
